# Leukonychie durch Lasertherapie einer Onychomykose

**DOI:** 10.1007/s00105-025-05536-7

**Published:** 2025-07-23

**Authors:** Frances Kuropka, Uwe Paasch, Jan Christoph Simon, Anna-Theresa Seitz

**Affiliations:** Klinik und Poliklinik für Dermatologie, Venerologie und Allergologie, Universitätsklinikum Leipzig, Philipp-Rosenthal-Str. 23, 04103 Leipzig, Deutschland

**Keywords:** Optische Kohärenztomographie, Neodym-YAG-Laser, Thermischer Effekt, Selektive Photothermolyse, Thermische Laserbehandlung

## Anamnese

Eine 69-jährige Patientin stellte sich in der ambulanten Sprechstunde der Klinik und Poliklinik für Dermatologie, Venerologie und Allergologie des Universitätsklinikums Leipzig AöR (Anstalt des öffentlichen Rechts) mit seit 12 Monaten klinisch bestehender Onychomykose an beiden Großzehennägeln vor. Eine extern durchgeführte topische Therapie mit Amorolfin-Hydrochlorid-haltigem Nagellack sowie einem Ciclopirox-haltigen Nagellack jeweils über mehrere Monate und eine Laserbehandlung (weitere Angaben zu der genauen Laservortherapie waren nicht eruierbar) hatten keine Befundbesserung ergeben.

## Befund

**Abb. 1:**
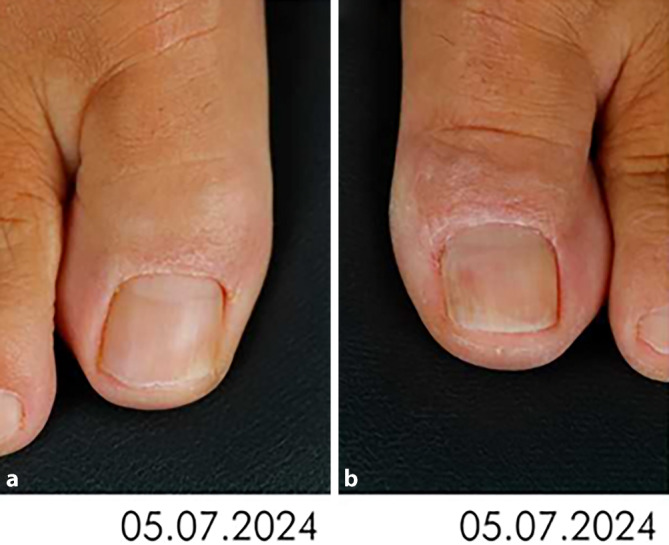
Klinische Bilder. **a** Rechte Großzehe der Patientin, Nagelplatte bei Erstvorstellung in der Klinik und Poliklinik für Dermatologie, Venerologie und Allergologie des Universitätsklinikums Leipzig. **b** Linke Großzehe der Patientin, Nagelplatte bei Erstvorstellung in der Klinik und Poliklinik für Dermatologie, Venerologie und Allergologie des Universitätsklinikums Leipzig

Bei Erstvorstellung wies die Nagelplatte der rechten Großzehe eine gelbliche Verfärbung im Randbereich der distalen, lateralen Nagelkante sowie einen verminderten Glanz auf (Abb. 1a, b). Der Nagel des linken Digitus pedis I zeigte neben den oben genannten Veränderungen außerdem eine dezente Verdickung der Nagelplatte. Die Haut beider Füße wies keine pathologischen Veränderungen auf, die Nagelplatten aller restlichen Zehen beider Füße zeigten sich altersentsprechend unauffällig.

## Diagnose

**Abb. 2:**
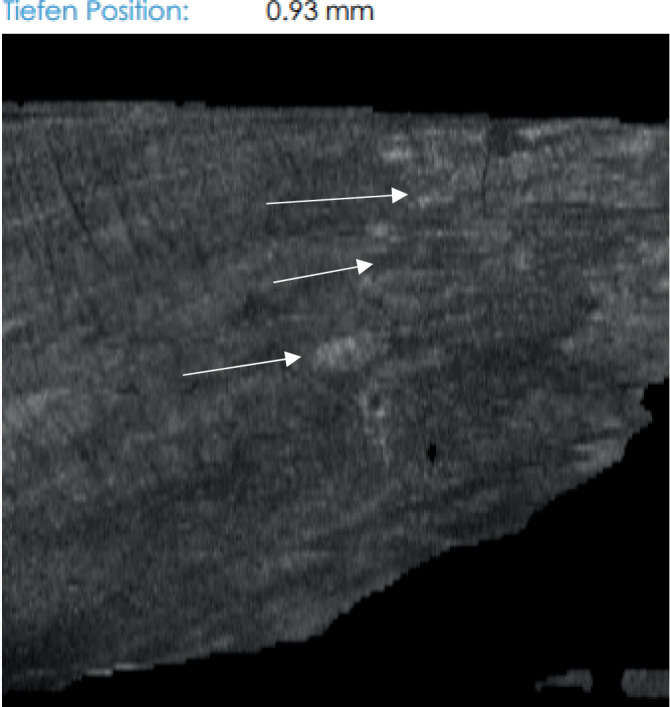
Befunddarstellung vom 05.07.2024 der optischen Kohärenztomographie (VivoSight Dx®-System der Firma Michelson Diagnostics Ltd, Kent, UK), Nagelplatte der linken Großzehe, zur Beurteilung der Onychomykosediagnose. *Weiße Pfeile* markieren die Pilzhyphen

Die durchgeführte Pilzkultur des subungualen Nagelmaterials der linken Großzehe ergab keinen Erregernachweis. Mittels optischer Kohärenztomographie (VivoSight Dx®-System der Firma Michelson Diagnostics Ltd, Kent, UK) konnten Hyphen dargestellt werden (Abb. 2). In Zusammenschau der Befunde konnte die Diagnose einer Onychomykose beider Großzehennägel gestellt werden.

## Therapie und Verlauf

**Abb. 3:**
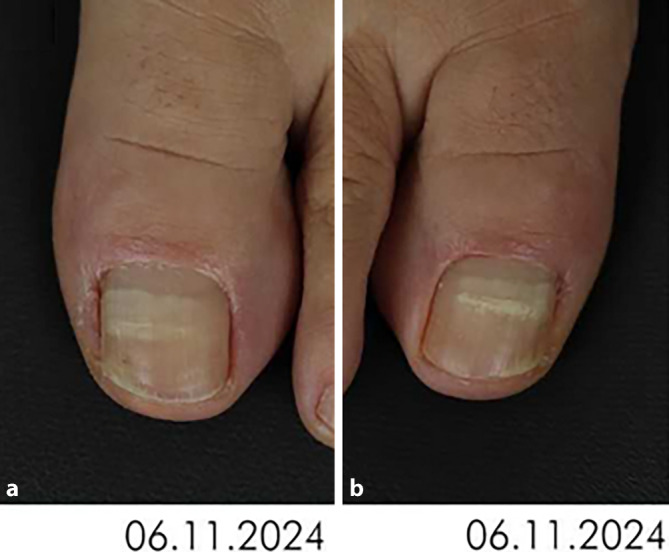
Klinische Bilder. **a** Nagelplatte der linken Großzehe mit weißlichen Verfärbungen nach Laserbehandlung. **b** Nagelplatte der rechten Großzehe mit weißlichen Verfärbungen nach Laserbehandlung

Parallel zur Weiterführung der topischen Therapie mittels Ciclopirox-haltigem Nagellack wurde der Patientin eine Pulstherapie mit Itraconazol 100 mg 2‑mal täglich (Einnahme täglich über 1 Woche, dann 3 Wochen Pause und anschließend erneute Einnahme über 1 Woche) verordnet. Zusätzlich zur topischen und systemischen Therapie erfolgte eine thermische Laserbehandlung mittels 1064-nm-Neodym-YAG-Laser (Mydon®, Alma Lasers GmbH, Nürnberg, Deutschland) mit einer Spotgröße von 5 mm, einer Pulsdauer von 40 ms und einer Fluenz von 120 J/cm^2^. Insgesamt erfolgten 4 Behandlungen beider Großzehennägel jeweils im Abstand von 4 Wochen. Bei der Vorstellung zur dritten Laserbehandlung zeigte sich erstmals im Bereich beider Nagelplatten der rechten und linken Großzehe eine streifenförmige Weißfärbung der Nagelplatte (Leukonychia striata) (Abb. 3a, b). Nach Durchführung von 4 Laserbehandlungen wurden diese sowie die systemische Therapie beendet. Die topische Therapie mit Ciclopirox-haltigem Nagellack wurde fortgesetzt.

## Diskussion

Die Onychomykose ist eine weitverbreitete Infektion, die das Nagelorgan betrifft. Besonders häufig sind dabei die Zehennägel betroffen. Diese Art von Pilzinfektion wird hauptsächlich durch Dermatophyten wie *Trichophyton rubrum* und *Trichophyton mentagrophytes* verursacht. Die Onychomykose stellt oft eine Herausforderung für die Diagnosestellung und Behandlung dar. Noch immer sind mit der Diagnose Onychomykose teils erhebliche Kosten, langwierige Behandlungszeiträume, Therapieversagen und hohe Rezidivraten verbunden. Die optische Kohärenztomographie kann im Rahmen der Diagnostik unterstützende Hilfe leisten. Als nichtinvasive Untersuchungsmethode ermöglicht sie eine schmerzfreie Anwendung und die Befunddarstellung in Echtzeit. Mittels optischer Kohärenztomographie ist jedoch keine Differenzierung der Pilzarten möglich, wie sie mittels mykologischer Kultur bzw. Polymerasekettenreaktionsdiagnostik erfolgt [8].

In unserem Fall konnte mittels Kultur kein Erregernachweis erfolgen, was am ehesten auf die antimykotische Vorbehandlung der Nägel zurückzuführen war. Die mykologischen Kulturen stellen zwar den Goldstandard in der Diagnostik dar, können aber unter anderem aufgrund vorheriger antimykotischer Behandlungen zu falsch negativen Ergebnissen führen [5], da die kulturelle Anzucht auf vitale Pilzelemente angewiesen ist [5]. Bei Befall der distalen Nagelanteile wie bei unserer Patientin sind die Pilzelemente häufig bereits abgestorben. Bei vorbehandelten Personen kann die Vitalität der Pilzelemente außerdem durch die antimykotische Therapie beeinflusst sein. Es ist daher wichtig, die Ergebnisse der Kulturen im Kontext der klinischen Befunde sowie etwaiger Fehlerquellen bei der Materialentnahme zu betrachten. Alternativen wie der elektronenmikroskopische Nachweis oder die Immunfluoreszenz könnten in solchen Fällen zusätzliche diagnostische Werkzeuge darstellen [9]. Die Anwendung der optischen Kohärenztomographie kann neben der Diagnostik auch für die Verlaufskontrolle unter Therapie eingesetzt werden. Gemäß der aktuellen Leitlinie „Onychomykose“ ist die vollständige und sichere Beseitigung des Erregers das oberste Ziel [5].

Der Behandlungserfolg sollte dabei durch eine negative Kontrolluntersuchung, z. B. in Form einer negativen Kultur, bestätigt werden. Dabei gilt es, auf einen zeitlichen Abstand zwischen Therapieende und der Materialgewinnung für die Kultur zu achten. Eine Kontrolluntersuchung direkt nach Therapieende könnte – wie in unserem Fall – zu einem falsch negativen Befund führen [5]. Zur weiteren Befundkontrolle nach abgeschlossener Behandlung – mit dem Wissen, dass Kulturen falsch negative Ergebnisse liefern können – könnte hier zusätzlich die optische Kohärenztomographie zum Einsatz kommen. Diese war bei unserer Patientin in der Verlaufskontrolle nicht erfolgt. Ein weiteres Ziel in der Leitlinie sind klinisch weitgehend gesunde Nägel (definiert meist als < 5–10 % Restveränderung am distalen Nagelrand) sowie die Verhinderung weiterer Übertragungen bzw. die Unterbrechung von Infektionsketten [5].

Gemäß der aktuellen Leitlinie „Lasertherapie der Haut“ kann der Einsatz von Hitzeapplikation mittels 1064-nm-Laser in Kombination mit topischer oder systemischer antimykotischer Therapie erfolgen [4]. Die Wirksamkeit einer alleinigen Laserbehandlung bei Onychomykose wird als nicht ausreichend angesehen [4, 5]. Das Prinzip der Laserbehandlung besteht hauptsächlich in der Erwärmung des Nagels sowie des Nagelbettes, wobei im Nagel mittlere Temperaturen von etwa 50 °C erreicht werden [4, 5]. Durch die Hitze kommt es zur Schädigung des Pilzes im Nagelorgan. Der befallene Nagel wird während einer Lasersitzung flächig in Gänze behandelt. Die hohen Temperaturen im Nagel und am Nagelbett unter der Lasertherapie verursachen Schmerzen, was therapielimitierend sein kann [4, 5]. Gemäß Leitlinie soll bei der Laserbehandlung der Zehennägel ausdrücklich keine Anästhesie angewendet werden, da die Schmerzempfindung als limitierend wahrgenommen werden soll [4]. Prinzipiell kann bei einem 1064-nm-Neodym-YAG-Laser das „optische Fenster“ der Haut genutzt werden, um sehr tief – mehrere Millimeter – einzudringen [5]. Das optische Fenster der Haut bezeichnet einen Wellenlängenbereich, in dem die Absorption unter anderem durch Wasser, Melanin sowie andere Hautbestandteile relativ gering ist, wodurch das Laserlicht in diesem Bereich tiefer in das Gewebe eindringen kann, ohne zu viel Energie in der Haut selbst zu verlieren. Für die Haut liegt dieses optische Fenster etwa im Bereich von 600–1300 nm. Am transluzenten und auch infizierten Nagel wird davon ausgegangen, dass die Penetrationstiefe zwar reduziert ist, aber entsprechend der Leitlinie auf jeden Fall ausreichen sollte, um den Erreger im Bereich des Nagelbettes zu erreichen [5]. Dies gilt besonders für den langgepulsten 1064-nm-Neodym-YAG-Laser. Im direkten Vergleich sind die kurzgepulsten 1064-nm-Neodym-YAG-Laser hier weniger wirksam [1, 7].

Vor Laserbehandlung bei Onychomykose muss eine Aufklärung über alternative Behandlungsmöglichkeiten, mögliche Nagelverfärbungen, ein Nichtansprechen, Schmerzen oder Nageldystrophie erfolgen [8]. Eine Nagelverfärbung kann als sog. Leukonychie eine mögliche Nebenwirkung darstellen. Darunter versteht man eine punktförmige, gestreifte oder totale Weißfärbung der Fuß- und/oder Fingernägel [6]. Eine Leukonychie entsteht wahrscheinlich durch eine parakeratotische Verhornung, die durch eine Irritation im Bereich des Nagels, genauer der Nagelmatrix, verursacht wird. In unserem Fall stellt die thermische Schädigung im Rahmen der Lasertherapie die Irritation bzw. das Trauma dar, das im Bereich der Nagelmatrix die Verhornung der nagelbildenden Zellen beeinträchtigt und zu einer Veränderung des Lichtreflexes des Nagels führt [6]. Ein solcher klinischer Befund mit weißlicher Nagelverfärbung kann auch im Zusammenhang mit einer Onychomykose als Leukonychia mycotica auftreten und stellt in diesem Fall eine Pseudoleukonychie dar, bei der sich die Onychomykose als Infektion der Nageloberfläche im Sinne einer weißen superfiziellen Onychomykose präsentiert [5] – was von der hier beschriebenen Leukonychie unter Laserbehandlung zu unterscheiden ist. Bei unserer Patientin trat die Nagelverfärbung, die sich als 2 horizontale weißliche Linien präsentierte (s. Abb. 3a, b), im direkten zeitlichen Zusammenhang mit der erfolgten Laserbehandlung auf und präsentiert sich klinisch als Leukonychia striata.

Das Nagelwachstum, ausgehend von der Nagelmatrix, ist ein kontinuierlicher Prozess. Im Durchschnitt ist ein Längenwachstum von ungefähr 2–4 mm pro Monat zu verzeichnen [10]. Dieses Wachstum hängt von der Lokalisation des Nagels (Finger oder Zehen) sowie vom Alter des Menschen und damit verbundenen physiologischen Eigenschaften wie Durchblutung, Ernährung und mechanischer Belastung ab. Die in unserem Fall aufgetretenen Nagelverfärbungen zeigten sich im zeitlichen Verlauf erstmalig nach der dritten Laserbehandlung. Dabei lag zwischen zweiter und dritter Laserbehandlung ein Zeitraum von 3 Wochen, zwischen dritter und vierter ein Zeitraum von 4 Wochen. Der direkte zeitliche Zusammenhang mit der Laseranwendung macht eine Leukonychie als Nebenwirkung der Lasertherapie wahrscheinlich, die sich pathophysiologisch durch die thermischen Effekte auf das umliegende Gewebe erklären lässt. Als weiterer Auslöser der Leukonychie sollte die Einnahme von Itraconazol in Erwägung gezogen werde. Eine solche Nebenwirkung wird in der Fachinformation sowie in der Literatur jedoch bisher nicht beschrieben [2]. Als weitere Differenzialdiagnose muss die Pseudoleukonychie erwogen werden, bei der die Nägel eine auffällige, weiße Verfärbung aufweisen, die jedoch nicht auf einer Erkrankung des Nagels oder der Nagelmatrix beruht. Diese Verfärbung kann durch verschiedene Faktoren verursacht werden, z. B. durch das Tragen von Nagellack oder durch bestimmte chemische Einflüsse. Im Gegensatz zur echten Leukonychie, die auf eine Schädigung oder Erkrankung der Nagelmatrix hinweist, ist die Pseudoleukonychie in der Regel harmlos und vorübergehend. In unserem Fall stellt die thermische Wirkung im Gewebe als Trauma der Nagelmatrix die Ursache für die Leukonychie dar. Um eine klare Unterscheidung zwischen Pseudoleukonychie und echter Leukonychie zu treffen, könnte eine wiederholte optische Kohärenztomographie hilfreich sein.

Als wahrscheinliche Ursache für die aufgetretenen Nagelverfärbungen bei unserer Patientin sehen wir in Zusammenschau aller Aspekte die thermischen Effekte des 1064-nm-Neodym-YAG-Lasers. Während der Laserbehandlung des gesamten Nagels wird die Energie der optischen Strahlung in Form von Wärme von den Zielstrukturen aufgenommen und auch an das umgebende Gewebe abgegeben. Das lokale Erhitzen führt somit zur Zerstörung der Zielstrukturen, kann jedoch im umliegenden Gewebe möglicherweise eine temporäre Schädigung der Nagelmatrix verursachen.

Analog zu der hier angenommenen temporären thermischen Schädigung der Nagelmatrix, welche die Leukonychie bedingt, wird ein ähnlicher Effekt auch als Nebenwirkung im Zusammenhang mit einer systemischen Zytostatikagabe beobachtet [3].

## Fazit für die Praxis

- Unter der Anwendung eines Neodym-YAG-Lasers zur thermischen Laserbehandlung bei Onychomykose können weißliche Nagelverfärbungen auftreten.
- Es gilt, dies unter Therapie klinisch zu beobachten und ggf. die Therapie zu beenden.
- Für einen optimalen Behandlungserfolg der Onychomykose unter Lasertherapie ist die begleitende Anwendung einer topischen oder systemischen Therapie notwendig.

## References

[1] Hochman LG (2011) Laser treatment of onychomycosis using a novel 0.65-millisecond pulsed Nd:YAG 1064-nm laser. J Cosmet Laser Ther 13:2–5 21250792 10.3109/14764172.2011.552616

[2] https://www.fachinfo.de/fi/pdf/009409/itraconazol-abz-100-mg-hartkapseln. Zugegriffen: 26. März 2025

[3] https://register.awmf.org/de/leitlinien/detail/032-054OL. Zugegriffen: 26. März 2025

[4] https://register.awmf.org/assets/guidelines/013-095l_S2k_Lasertherapie-der-Haut_2022-03.pdf. Zugegriffen: 26. März 2025

[5] https://www.awmf.org/leitlinien/detail/ll/013-003.html. Zugegriffen: 26. März 2025

[6] Iorizzo M, Starace M, Pasch MC (2022) Leukonychia: What Can White Nails Tell Us? Am J Clin Dermatol 23:177–193 35112320 10.1007/s40257-022-00671-6 PMC8809498

[7] Kozarev J, Vizintin Z (2010) Novel laser therapy in treatment of onychomycosis. J Laser Health Acad 1:1–8

[8] Rothmund G, Sattler EC, Kaestle R et al (2012) Confocal laser scanning microscopy as a new valuable tool in the diagnosis of onychomycosis—comparison of six diagnostic methods. Mycosis 56:47–55 10.1111/j.1439-0507.2012.02198.x 22524550

[9] Vennewald I et al (2008) Topography of dermatophyte infection in onychomycosis—fluorescent and electron microscopic investigations. Med Mycol 15:7–12

[10] Wollina U et al (2016) Diagnostik und Therapie von Nagelerkrankungen. Dtsch Ärzteblatt Int 113:509–518

